# Generalized Landauer Bound for Information Processing: Proof and Applications

**DOI:** 10.3390/e24111568

**Published:** 2022-10-31

**Authors:** Neal G. Anderson

**Affiliations:** Department of Electrical & Computer Engineering, University of Massachusetts Amherst, Amherst, MA 01003-9292, USA; anderson@ecs.umass.edu

**Keywords:** Landauer’s Principle, physics of information and computation, irreversibility

## Abstract

A generalized form of Landauer’s bound on the dissipative cost of classical information processing in quantum-mechanical systems is proved using a new approach. This approach sidesteps some prominent objections to standard proofs of Landauer’s bound—broadly interpreted here as a nonzero lower bound on the amount of energy that is irreversibly transferred from a physical system to its environment for each bit of information that is lost from the system—while establishing a far more general result. Specializations of our generalized Landauer bound for ideal and non-ideal information processing operations, including but not limited to the simplified forms for erasure and logical operations most familiar from the literature, are presented and discussed. These bounds, taken together, enable reconsideration of the links between logical reversibility, physical reversibility, and conditioning of operations in contexts that include but are far more general than the thermodynamic model systems that are most widely invoked in discussions of Landauer’s Principle. Because of the strategy used to prove the generalized bounds and these specializations, this work may help to illuminate and resolve some longstanding controversies related to dissipation in computation.

## 1. Introduction

Landauer’s Principle (LP) posits a fundamental link between information and the physical systems that bear and process it. Since its introduction in 1961 [1], this qualitative principle and its quantitative expression as the Landauer Bound (LB) or Landauer Limit (LL) have been refined, restated, formalized and interpreted in a variety of ways 1. While some hew more closely to Landauer’s original argument than others, virtually all recastings of (the “energetic form” of) LP express the same essential claim: that the loss of information from a physical system in contact with a thermal environment at temperature *T* necessarily dissipates at least $k_{B}T\ln\left(2\right)$ of energy into that environment for each bit of information lost from the system, where $k_{B}$ is Boltzmann’s constant. Formally, this is expressed as the inequality

$$\Delta E\geq k_{B}T\ln\left(2\right)I_{er}$$

where ΔE is the amount of dissipated energy and $I_{er}$ is the amount of information erased. This is the Landauer Bound (LB).

As simple and straightforward as it may appear, the Landauer bound is open to a wider variety of interpretations than one might expect. This, together with the fact that it is often unclear which interpretation a given author is adopting or defending, has contributed to a controversy over the validity of LP that has persisted for decades 2. These interpretive ambiguities and controversies have multiple sources, four of which are particularly relevant to the present work. First, while the mathematical expression of any physical relationship is obviously ambiguous without clear and precise definition of all relevant physical quantities, processes, and assumptions, the literature on LP is varied in how clearly, precisely, and completely authors declare, specify, and justify their choices. There are more options than might meet the eye—for the dissipated energy *E* and erased information $I_{er}$, for entropy and other important intermediate quantities that arise in proofs and demonstrations of the LB, and for the very notion of erasure and definition of an erasure operation. Second, various authors consider LP in different physical settings. Many studies provide concrete demonstrations of LP for historically familiar model systems—particularly the one-molecule gas in a partitioned cylinder (the Szilard engine) or its quantum analog in the form of the double potential well—and on standard physical relations that apply to those models. While such demonstrations are appealing in their concreteness, they are insufficient to establish LP as a general and necessary consequence of physical law. Third, some authors implicitly interpret the LB—an inequality—to be a claim that the $k_{B}T\ln\left(2\right)$ of environmental heating per erased bit is an achievable lower limit while others regard it simply as an inviolable lower bound that carries no claim of achievability, and the intention is not always clear. Fourth and finally, methodological objections have been leveled at various proofs, demonstrations, and arguments in support of LP. If sustained, these objections would invalidate LP or, as Norton [6] has put it, leave us “without a cogent justification of Landauer’s Principle”.

As long as controversy over LP persists, its status—and the essential connection it provides between information and physics—will remain unresolved. This will further continue to infect debates over related issues like Maxwell’s Demon [2,4] and reversible computing 3. This state of affairs motivates pursuit of clear and precise proofs of the LB that can establish it as a direct, transparent, and general consequence of physical law and that address or sidestep objections and concerns that have hindered its resolution and broad acceptance.

In this work, we offer such a proof. We prove a very general form of the LB—drawing from little more than lawful quantum dynamics and established entropic inequalities—and show how it can be specialized to a variety of ideal and non-ideal classical information processing operations implemented in closed quantum systems interacting with thermal environments. (Here we will say that a classical information-processing operation is ideal if and only if the physical states that encode all distinct computational inputs are perfectly distinguishable from one another and that the same is true of the physical states that encode all computational outputs.) Because of the way in which the dissipated energy is defined in our “generalized Landauer bound” (GLB), and because of the way in which the lower bound on this dissipated energy is proved, common objections to existing proofs of LB are sidestepped. These include prominent objections related to the construction of ensembles and the combining or equating of physical and information-theoretic entropies. It also provides insight into the link between logical and physical reversibility in physical implementations of information processing operations.

The paper is organized as follows. In Section 2, we introduce and precisely characterize the very general physical setting in which we obtain our GLB and define the “trial-averaged” energy quantity to which this bound pertains. We then present our GLB, define all associated quantities and processes, and discuss its application to the physical implementation of arbitrary *M*-input to *N*-output (*M*-to-*N*) information processing operations in scenarios where *M* initial input states of a system are overwritten by *N* final output states of that system. Such scenarios accommodate the model systems and processes that are almost ubiquitously employed in studies of Landauer’s Principle, such as Szilard engines, coupled potential-well arrays, and field-coupled logic implementations (e.g., [9]), which interact with thermal environments and exchange heat and work with their surroundings as they process information 4. Next, we specialize the GLB to ideal and non-ideal Landauer erasure (i.e., “erasure-by-reset”) processes (*M* > *N* = 1), including the simplified forms familiar from the literature that apply under standard assumptions about the erasure scenario and protocol. We then specialize the GLB to logically irreversible (*M* > *N* > 1) and logically reversible (*M* = *N* ≥ 1) information processing operations. In Section 3, we then present our proof the GLB. This proof substantially generalizes an approach employed in Ref. [5] to lower bound the dissipative cost of ideal Landauer erasure processes only. Finally, in Section 4, we summarize our work and highlight the features that distinguish it from other studies of Landauer’s Principle and that can help to resolve associated controversies.

## 2. Generalized Landauer Bound and Applications

### 2.1. Setup

We consider a family of *M* individual physical processes

$${\hat{\rho }}_{i}^{S}⊗{\hat{\rho }}_{th}^{E}\to {\hat{\rho }}_{i}^{{\mathrm{SE}}^{\prime }}\;i=0,1,\ldots M-1$$

on a globally closed system-environment composite SE, each of which begins with the system S in one of the *M* initial states ${\hat{\rho }}_{i}^{S}$ and the environment E in the thermal state

$${\hat{\rho }}_{th}^{E}=\exp\left\{-{\hat{H}}^{E}/k_{B}T\right\}.$$

Here ${\hat{H}}^{E}$ is the environment Hamiltonian, $k_{B}$ is Boltzmann’s constant, and *T* is the temperature. The symbol $\hat{\rho }$ generically denotes quantum mechanical density operators, with superscripts used to label density operators representing states of the global composite SE and local (reduced) subsystem states of (i.e., of S or E). Unprimed and primed superscript labels on density operators denote initial states (at time t=0) and final states (at time $t=t_{op}$), respectively.

Consistent with Schrodinger evolution of globally closed composite systems, the initial and final states of SE are, for the *i*-th process, related as

(1) $${\hat{U}}_{i}\left({\hat{\rho }}_{i}^{S}⊗{\hat{\rho }}_{th}^{E}\right){\hat{U}}_{i}^{†}={\hat{\rho }}_{i}^{{\mathrm{SE}}^{\prime }}$$

where ${\hat{U}}_{i}$ is the unitary operator

(2) $${\hat{U}}_{i}=T\left\{\exp\left[-\frac{i}{\hbar }\int_{0}^{t_{op}}{\hat{H}}_{i}^{\mathrm{SE}}\left(t\right)dt\right]\right\}.$$

Here, ${\hat{H}}_{i}^{\mathrm{SE}}\left(t\right)$ is the global Hamiltonian for the *i*-th process and T denotes the Dyson time-ordering operator. We specifically consider global Hamiltonians of the form

(3) $${\hat{H}}_{i}^{\mathrm{SE}}\left(t\right)=\left({\hat{H}}^{S}+{\hat{H}}^{E}+{\hat{H}}_{int}^{\mathrm{SE}}+V_{i}^{S}\left(t\right)\right)$$

where ${\hat{H}}^{S}$ and ${\hat{H}}^{E}$ are the system and environment self-Hamiltonians—defined on fixed Hilbert (state) spaces of S and E, respectively, —${\hat{H}}_{int}^{\mathrm{SE}}$ is the system-environment interaction Hamiltonian, and $V_{i}^{S}\left(t\right)$ is a finite time-dependent potential energy applied to the system for the *i*-th process (i.e., for the initial system state ${\hat{\rho }}_{i}^{S}$). We have allowed, pending later specialization, that each of the *M* independent processes is guided by application of a different time-dependent process potential $V_{i}^{S}\left(t\right)$ to system S, so the differences in the ${\hat{U}}_{i}$ for the different processes stem exclusively from differences in the *M* processes potentials.

This is a very general description of a set of *M* independent processes resulting from lawful Schrodinger evolution of a closed quantum system interacting with a(n initially) thermal bath at temperature *T*, with each process 5 characterized by its own initial system state ${\hat{\rho }}_{i}^{S}$ and applied potential $V_{i}^{S}\left(t\right)$ that takes the initial system state ${\hat{\rho }}_{i}^{S}$ to a final state ${\hat{\rho }}_{i}^{S^{\prime }}=Tr_{E}\left[{\hat{\rho }}_{i}^{{\mathrm{SE}}^{\prime }}\right]$. No assumptions have been made at this point about the mutual distinguishability of the initial states or of the final states of the *M* processes. Nor have any assumptions been made about the forms of any of the initial system states, final system states, or final environment states of these processes.

Looking ahead to specialization of this description to various information processing operations, we will take the initial and final states of *M* processes to be physically encoded inputs and outputs of *M*-input-to-*N*-output classical information processing operations implemented in S via those *M* physical processes. Our ultimate focus will be lower bounds on the amount of energy transferred to the environment in ideal and imperfect erasure operations and in logical operations.

Returning to the general description, from which these specialized results will be obtained, we write the change in the environment energy for the *i*-th process as

(4) $$\Delta \langle E_{i}^{E}\rangle =\langle E_{i}^{E^{\prime }}\rangle -\langle E_{i}^{E}\rangle$$

where $\langle E_{i}^{E}\rangle$ and $\langle E_{i}^{E^{\prime }}\rangle$ are the initial and final expected values of the environment energy, respectively, as given by

$$\langle E_{i}^{E}\rangle =Tr_{E}\left[{\hat{\rho }}_{th}^{E}{\hat{H}}^{E}\right]\equiv \langle E_{th}^{E}\rangle$$

and

$$\langle E_{i}^{E^{\prime }}\rangle =Tr_{E}\left[{\hat{\rho }}_{i}^{E^{\prime }}{\hat{H}}^{E}\right]\;with{\hat{\rho }}_{i}^{E^{\prime }}=Tr_{S}\left[{\hat{\rho }}_{i}^{{\mathrm{SE}}^{\prime }}\right].$$

Note that, because the composite SE is closed and the process potentials $V_{i}^{S}\left(t\right)$ act only on the system S, such changes in the energy of E result exclusively from exchange of energy with S. The energy bounds we obtain are specifically lower bounds on the average

(5) $$\langle \Delta \langle E_{i}^{E}\rangle \rangle =\sum_{i=0}^{M-1}p_{i}\Delta \langle E_{i}^{E}\rangle$$

of the (expected values of) environment energy changes for the *M* independent processes. This corresponds to the kind of average one would straightforwardly obtain from measurements of $\Delta \langle E_{i}^{E}\rangle$ over large number of individual trials, a fraction $p_{i}$ of which begin with the joint system prepared in the *i*-th initial state ${\hat{\rho }}_{i}^{S}⊗{\hat{\rho }}_{th}^{E}$ and evolve under applied potential $V_{i}^{S}\left(t\right)$. We emphasize this because proofs and demonstrations of Landauer-like bounds generally lower bound the dissipation for a single process involving the “average state” of the ensemble of *M* states, without explicitly recognizing that any individual trial evolves only one of the *M* initial states (i.e., one of the “logical inputs”) and demonstrating that use of such an average state to obtain dissipation bounds is a valid surrogate for averaging dissipation over the *M* trials. Since we consider bounds on the “trial-averaged” change in the environment energy $\langle \Delta \langle E_{i}^{E}\rangle \rangle$ in this work, the results presented here are immune to concerns related to this practice. We present, discuss, and specialize our generalized bound in the remainder of this section, and provide the associated proof in Section 3.

### 2.2. The Generalized Landauer Bound

The central result of this work, from which all others follow as specializations, is proof of a fundamental lower bound on the average environment energy increase $\langle \Delta \langle E_{i}^{E}\rangle \rangle$ over the *M* independent processes described above. Such a bound is obtained under two different assumptions about the set of *M* time-dependent potentials $V_{i}^{S}\left(t\right)$ associated with these processes, the second of which is what we call the generalized Landauer bound.

The first bound—which we will call the “conditional energy bound”—makes no assumption about the relationships between the *M* process potentials $V_{i}^{S}\left(t\right)$. 6 The conditional energy bound, proven in Section 3.1 as a stepping stone to proof of the unconditional energy bound (the GLB), is simply as follows:

- **Conditional Energy Bound:** The energy change in the environment E, averaged over the *M* processes, is lower bounded as
  (6) $$\langle \Delta \langle E_{i}^{E}\rangle \rangle \geq -k_{B}T\ln\left(2\right)\sum_{i}p_{i}\Delta S_{i}^{S}$$
  where
  (7) $$\Delta S_{i}^{S}=S\left({\hat{\rho }}_{i}^{S^{\prime }}\right)-S\left({\hat{\rho }}_{i}^{S}\right)$$
  is the increase in the von Neumann entropy—defined for any density operator $\hat{\rho }$ as $S\left(\hat{\rho }\right)\equiv -Tr\left[\hat{\rho }\log_{2}\hat{\rho }\right]$—of system S for the *i*-th processes.

Without granting any thermodynamic significance to the von Neumann entropy, we note that the “TΔS” form of this bound has a thermodynamic ring to it. Formally, it simply says that, for each of the *M* processes, any reduction in the von Neumann entropy of a system increases the energy of a thermal bath with which it is in contact by an amount that is never smaller than the product of the system entropy decrease and the initial bath temperature (with the $k_{B}\ln\left(2\right)$ factor accommodating differences in “thermodynamic units” and “information theoretic” units of entropy).

We can also observe both a similarity and a striking difference between the conditional energy bound and Landauer’s lower bound on the energy cost of irreversible information loss. The similarity is the presence of the signature $k_{B}T\ln\left(2\right)$ proportionality constant between energy and information in the LB. The striking difference, however, is the complete absence from this bound of any quantity that could rightfully be interpreted as information loss associated with an *M*-input information processing operation. 7 Finally, we note that nothing in the conditional energy bound precludes restoration of any or all of the evolved states ${\hat{\rho }}_{i}^{S^{\prime }}$ of S to their initial states ${\hat{\rho }}_{i}^{S}$ by application of an appropriately chosen potential to the system after time $t_{op}$, e.g., a time-reversed version of $V_{i}^{S}\left(t\right)$. Note that this is the case even if the *M* final states ${\hat{\rho }}_{i}^{S^{\prime }}$ are not mutually distinguishable.

As shown below, however, an additional term in the energy bound—one that does have information-theoretic significance and is directly identifiable with irreversible system-to-environment energy flow—emerges under a one straightforward condition on the set of *M* process potentials: that the same process potential $V^{S}\left(t\right)$ is unconditionally applied to the system whatever the initial state (i.e., $V_{i}^{S}\left(t\right)=V^{S}\left(t\right)$∀i). This unconditional energy bound—which we will call the “generalized Landauer bound” and prove in Section 3.2—is as follows:

- **Generalized Landauer Bound:** The energy change in the environment E, averaged over the *M* processes, is lower bounded as
  (8) $$\langle \Delta \langle E_{i}^{E}\rangle \rangle \geq -k_{B}T\ln\left(2\right)\Delta \chi^{S}-k_{B}T\ln\left(2\right)\sum_{i}p_{i}\Delta S_{i}^{S}$$
  where
  (9) $$\Delta \chi^{S}=\chi^{S^{\prime }}-\chi^{S}$$
  is the change in the Holevo information [10] for the ensemble of *M* system states—defined as
  (10) $$\chi =S\left(\sum_{i}p_{i}{\hat{\rho }}_{i}\right)-\sum_{i}p_{i}S\left({\hat{\rho }}_{i}\right)$$
  for any ensemble $\epsilon =\{p_{i},{\hat{\rho }}_{i}\}$ of density operators—and $\Delta S_{i}^{S}$ is the average self-entropy change for the *i*-th process as defined in (7).

The unconditional energy bound (8) is identical to the conditional bound (6) apart from the emergence of the term proportional to the change in Holevo information of the ensemble of system states resulting from the operation. This term, which necessarily results from the unconditional nature of the operation, does have information theoretic significance in the present context. Specifically, the Holevo information of an ensemble of system states is an established upper bound on the accessible information—the maximum amount of classical information encoded in the ensemble that can be retrieved from physically possible quantum measurements on S. The Holevo information for ensemble $\epsilon =\{p_{i},{\hat{\rho }}_{i}\}$ is upper bounded as $\chi \leq H(\left\{p_{i}\right\})$, where $H\left(\left\{p_{i}\right\}\right)=-\sum_{i}p_{i}\log_{2}p_{i}$ is the Shannon entropy (or Shannon “information”) [11] of probability mass function $\left\{p_{i}\right\}$. Equality is achieved in the upper bound when all of the states ${\hat{\rho }}_{i}$ in the ensemble are mutually distinguishable, i.e., when they have support on disjoint subspaces of the system’s state space.

Two aspects of the “information term” $-k_{B}T\ln\left(2\right)\Delta \chi^{S}$ in the generalized Landauer bound are of particular note. First, because Holevo information is nonincreasing under quantum operations, this term is nonnegative. (Specifically, this term is zero if the operation preserves Holevo information and is positive if Holevo information is lost from S.) Second, if $\Delta \chi^{S}$ is negative, so energy is transferred from system to environment under loss of Holevo information from the system, this energy transfer is irreversible. Application of any new time-dependent potential (or potentials) to the system after the conclusion of the operation (i.e., after $t=t_{op}$) would amount to another quantum operation, and, since quantum operations cannot increase Holevo information as mentioned above, there is no way to “get the information back” in S that has been lost to E a result of the operation. This is to say that the irreversible loss of Holevo information from a physical system is accompanied by irreversible transfer of energy from the system to the surrounding environment (i.e., energy dissipation), which is the essential claim of Landauer’s Principle stated precisely for scenarios that are substantially more general than those typically considered.

The fact that the proof of this unconditional bound (Section 3.2) differs from the proof of the conditional bound (Section 3.1) only in the lack of conditioning of the process potential on the inital state indicates that the physical origin of the energy cost of irreversible information loss is related to this lack of conditioning. 8 This is consistent with widespread identification of dissipation with a loss or erasure of “unknown data” or of information “without a copy”, although such terminology can be taken to suggest that the impersonal physical costs of physical processes depend on whether some sentient being knows the data being processed or whether there happens to be a copy of this data sitting around somewhere. Here we associate the energy cost of irreversible information loss with lack of conditioning of the operation on the initial state—unconditional application of the same potential $V^{S}\left(t\right)$ whatever the initial state rather than selective application of the potential $V_{i}^{S}\left(t\right)$ for the *i*-th initial state—which is intended to discourage such mysterious connotations while otherwise being similar in spirit. A sentient being performing the operation manually would have to “know” or have access to a “copy” of the initial state in order to act conditionally—to select the appropriate potential $V_{i}^{S}\left(t\right)$ for application—and would otherwise be left with no option other than to act unconditionally. If the operation were automated, as would be the case for some information processing device buried deep within a functioning computing system, conditional operation of this device would require a record of the initial state of S accessible to the mechanism that manipulates the device potential (e.g., in memory) 9. Such “internal conditioning” can be achieved via systems external to S that hold records of logical inputs and that interact continuously with S throughout execution of operations and, where applicable, their reversal (e.g., [9,13]).

### 2.3. Specialization: Energy Bounds for Landauer Erasure and Logical Operations

We now specialize the generalized Landauer bound for deterministic *M*-to-*N* classical information processing operations 10 implemented in S. By an *M*-to-*N* classical information processing operation, we mean a surjective *M*-to-*N* mapping (M≥N)—injective for M=N and non-injective for M>N—from *M* discrete input symbols $x_{i}\in \left\{x_{0},x_{1},\ldots ,x_{M}\right\}$ to *N* discrete output symbols $y_{j}\in \left\{y_{0},y_{1},\ldots ,y_{N}\right\}$. We take the initial and final states of *M* processes to be physical encodings of inputs and outputs of S, with input $x_{i}$ encoded by preparing S in state ${\hat{\rho }}_{i}$. Such operations generally include Landauer erasure (M>N with N=1), other logically irreversible operations (M>N with N>1), and logically reversible operations 11 (M=N).

To specialize the generalized Landauer bound for such operations, we note first that for an *M*-to-*N* operation there are *N* subsets of the *M* inputs, each subset associated with one of the *N* outputs. For the *j*-th of these subsets, every input $x_{i}$ in the subset maps into the same output $y_{j}$. Denoting the set of indices *i* that label inputs $x_{i}$ mapping into the *j*-th output as $i\in {\left\{i\right\}}_{j}$, and the final state of S that encodes output $y_{j}$ as ${\hat{\rho }}_{j}^{S^{\prime }}$, we can stipulate the following for a set of *M* physical state transformations

$${\hat{\rho }}_{i}^{S}⊗{\hat{\rho }}_{th}^{E}\to {\hat{\rho }}_{i}^{{\mathrm{SE}}^{\prime }}\;i=0,1,\ldots M-1$$

regarded as a physical implementation of this operation:

$$Tr_{E}\left[{\hat{\rho }}_{i}^{{\mathrm{SE}}^{\prime }}\right]={\hat{\rho }}_{j}^{S^{\prime }}foralli\in {\left\{i\right\}}_{j}\;j=0,1,\ldots N-1.$$

This condition simply states that for all inputs $x_{i}$ that map into output $y_{j}$, the initial physical states ${\hat{\rho }}_{i}^{S}$ of S that encode these inputs are transformed into the same final state ${\hat{\rho }}_{j}^{S^{\prime }}$ that encodes output $y_{j}$.

We now apply this condition to specialization of the generalized Landauer bound for Landauer erasure and logical operations.

#### 2.3.1. Landauer Erasure

In a Landauer erasure operation, all input (initial) states are “reset” to the same output (final) state. Thus, N=1 for any *M*-input Landauer erasure operation: there is only one “logical” output $y_{0}$ and one output state (or “reset” state)

$${\hat{\rho }}_{0}^{S^{\prime }}\equiv {\hat{\rho }}_{reset}^{S}.$$

The immediate consequence of this for the generalized Landauer bound is that there is no “ensemble” of output states. Rather, there is a single output state that obtains with unit probability for all input states so $\chi^{S^{\prime }}=0$. With this, we consider two Landauer erasure scenarios.

First, we make no assumption about the distinguishability of the input states, allowing that they may be imperfectly distinguishable. For this very general case, we have the following:

- **Landauer Bound for Erasure (General Encodings):** The average energy change in the environment E for an *M*-input Landauer erasure operation implemented in S, weighted by the relative frequencies $p_{i}$ with which the inputs $x_{i}$ are encoded, is lower bounded as
  (11) $$\langle \Delta \langle E_{i}^{E}\rangle \rangle \geq k_{B}T\ln\left(2\right)\chi^{S}-k_{B}T\ln\left(2\right)\sum_{i}p_{i}\Delta S_{i}^{S}$$
  where $\chi^{S}$ is the Holevo information of the input ensemble $\epsilon^{S}=\left\{p_{i},{\hat{\rho }}_{i}^{S}\right\}$.

Next, we assume that the input states ${\hat{\rho }}_{i}^{S}$ are mutually distinguishable, in which case we have the following:

- **Landauer Bound for Erasure (Distinguishable Encodings):** The average energy change in the environment E for an *M*-input Landauer erasure operation implemented in S, weighted by the relative frequencies $p_{i}$ with which the inputs $x_{i}$ are encoded, is lower bounded as
  (12) $$\langle \Delta \langle E_{i}^{E}\rangle \rangle \geq k_{B}T\ln\left(2\right)H\left(X\right)-k_{B}T\ln\left(2\right)\sum_{i}p_{i}\Delta S_{i}^{S}$$
  where H(X) is the Shannon entropy [11] of the (probability mass function $\left\{p_{i}\right\}$ of the) input random variable $X=\{p_{i},x_{i}\}$.

In both cases,

(13) $$\Delta S_{i}^{S}=S\left({\hat{\rho }}_{reset}^{S}\right)-S\left({\hat{\rho }}_{i}^{S}\right)$$

is the change in the von Neumann entropy of S for erasure of the *i*-th input.

Since $\chi^{S}\leq H\left(X\right)$, with equality for mutually distinguishable ${\hat{\rho }}_{i}$, the second bound holds for both general and distinguishable encodings. However, the bound for general encodings is tighter when the input states are imperfectly distinguishable, and quantifies the reduction in the lower bound on the physical cost of erasing the (smaller) amount of information that is actually encoded in S when the states are imperfectly distinguishable.

Further specialization to more familiar forms follows immediately from a series of three assumptions that are commonly made in the literature. The first assumption is that the self-entropies of all input states and the reset state are the same (i.e., $\Delta S_{i}^{S}=0$ for all *i*), in which case the second term vanishes and

$$\langle \Delta \langle E_{i}^{E}\rangle \rangle \geq k_{B}T\ln\left(2\right)H\left(X\right).$$

The second is that M=2, i.e., that a single binary digit is being erased, in which case $H\left(X\right)=H_{2}\left(p_{0}\right)$ where $H_{2}\left(p_{0}\right)=-p_{0}\log_{2}p_{0}-\left(1-p_{0}\right)\log_{2}\left(1-p_{0}\right)$ is the binary entropy function. This leaves

$$\langle \Delta \langle E_{i}^{E}\rangle \rangle \geq k_{B}T\ln\left(2\right)H_{2}\left(p_{0}\right).$$

The third and final assumption is uniform encoding, so $p_{0}=1-p_{0}=\frac{1}{2}$ and

$$\langle \Delta \langle E_{i}^{E}\rangle \rangle \geq k_{B}T\ln\left(2\right)$$

which translates directly to the oft quoted lower bound of $k_{B}T\ln\left(2\right)$ on the energy cost of erasing one bit. Recall that all of this is for unconditional erasure: the information term in the generalized Landauer bound—and thus in all of the specialized bounds given here—vanishes for conditional operations.

#### 2.3.2. Logically Irreversible Operations

In *M*-to-*N* logical operations, *M* inputs map into *N* outputs such that, for every output $y_{j}$, the subset of inputs $x_{i}$ with indices $i\in {\left\{i\right\}}_{j}$ map into output $y_{j}$. There are a number possible scenarios for physical implementation of any such operation, each with an energy cost bounded by its own specialization of the generalized Landauer bound. There are four classes of these scenarios, corresponding to cases where either, both, or neither of the set of *M* input states and the set of *N* output states are mutually distinguishable. Here we focus on the simplest case, where both the set of input states ${\hat{\rho }}_{i}^{S}$ and the set of output states ${\hat{\rho }}_{j}^{S^{\prime }}$ are mutually distinguishable.

For this case, the ensemble $\epsilon^{S^{\prime }}=\left\{p_{i},{\hat{\rho }}_{i}^{S^{\prime }}\right\}$ of evolved input states collapses to the ensemble $\epsilon^{S^{\prime }}=\left\{q_{j},{\hat{\rho }}_{j}^{S^{\prime }}\right\}$ of output states, where

(14) $$q_{j}=\sum_{i\in {\left\{i\right\}}_{j}}p_{i}$$

denotes the relative frequency of the *j*-th output. With this, and simplification of the Holevo information to Shannon entropy for ensembles of mutually distinguishable input states and mutually distinguishable output states, we have the following:

- **Landauer Bound for Logical Operations (Distinguishable Encodings):** The average energy change in the environment E for an *M*-input, *N*-output logical operation implemented in S, weighted by the relative frequencies $p_{i}$ with which the inputs $x_{i}$ are encoded, is lower bounded as
  (15) $$\langle \Delta \langle E_{i}^{E}\rangle \rangle \geq k_{B}T\ln\left(2\right)\left[H\left(X\right)-H\left(Y\right)\right]-k_{B}T\ln\left(2\right)\sum_{i}p_{i}\Delta S_{i}^{S}$$
  where H(X) and H(Y) are the Shannon entropies of the input and output random variables $X=\{p_{i},x_{i}\}$ and $Y=\{q_{j},y_{j}\}$, respectively. Equivalently 12
  (16) $$\langle \Delta \langle E_{i}^{E}\rangle \rangle \geq k_{B}T\ln\left(2\right)H\left(X|Y\right)-k_{B}T\ln\left(2\right)\sum_{i}p_{i}\Delta S_{i}^{S}$$
  where H(X|Y) is the conditional entropy for the correlated random variables *X* and *Y*.

Here

(17) $$\Delta S_{i}^{S}=S\left({\hat{\rho }}_{j}^{S^{\prime }}\right)-S\left({\hat{\rho }}_{i}^{S}\right)\;\mathrm{for}\;\mathrm{all}i\in {\left\{i\right\}}_{j}$$

is the change in the von Neumann entropy of S for transformation of the *i*-th input state. More familiar forms of the bounds (15) and (16)—forms that include only the first (information) term on the right-hand side—emerge under the common assumption that all input and output states have the same von Neumann entropy (so the second term vanishes).

## 3. Trial-Averaging Proof of the GLB

We now prove the generalized Landauer bound (8)—the lower bound on the average energy change $\langle \Delta \langle E_{i}^{E}\rangle \rangle$ of the environment over the *M* processes for unconditionally applied process potentials. The energy bound (6) for conditional process potentials is obtained along the way. The proof generalizes the approach taken in [5], which considered ideal Landauer erasure processes only 13.

### 3.1. Proof of the Conditional Energy Bound

We lower bound the trial-averaged environment energy increase

$$\langle \Delta \langle E_{i}^{E}\rangle \rangle =\sum_{i}p_{i}\Delta \langle E_{i}^{E}\rangle =\sum_{i}p_{i}\left(\langle E_{i}^{E^{\prime }}\rangle -\langle E_{i}^{E}\rangle \right)$$

by lower bounding the individual $\langle \Delta E_{i}\rangle$, noting that

(18) $$\Delta \langle E_{i}^{E}\rangle =\langle E_{i}^{E^{\prime }}\rangle -\langle E_{th}^{E}\rangle$$

for all *M* processes since each process begins with the environment in a thermal state. Here $\langle E_{th}^{E}\rangle =Tr_{E}\left[{\hat{\rho }}_{th}^{E}{\hat{H}}^{E}\right]$ is the energy of the environment when it is in a thermal state.

For the *i*-th process the environmental energy change is lower bounded as

(19) $$\Delta \langle E_{i}^{E}\rangle \geq k_{B}T\ln\left(2\right)\Delta S_{i}^{E}$$

by Partovi’s inequality [14], which applies precisely to the scenario at hand: a quantum system initially in a thermal state—here our environment E—that undergoes a change in entropy.

To lower bound the environmental entropy change $\Delta S_{i}^{E}$ for the *i*-th process, we make use of the fact that SE is a closed composite that undergoes unitary Schrodinger evolution from an initial state $\rho_{i}^{\mathrm{SE}}=\rho_{i}^{S}⊗{\hat{\rho }}_{th}^{E}$ to final state ${\hat{\rho }}_{i}^{{\mathrm{SE}}^{\prime }}={\hat{U}}_{i}{\hat{\rho }}_{i}^{\mathrm{SE}}{\hat{U}}_{i}^{†}$. Since von Neumann entropy is conserved under unitary evolution, we have

$$S\left({\hat{\rho }}_{i}^{{\mathrm{SE}}^{\prime }}\right)=S\left({\hat{\rho }}_{i}^{\mathrm{SE}}\right).$$

For the initial state of SE, we have

$$S\left({\hat{\rho }}_{i}^{\mathrm{SE}}\right)=S\left(\rho_{i}^{S}⊗{\hat{\rho }}_{th}^{E}\right)=S\left({\hat{\rho }}_{i}^{S}\right)+S\left({\hat{\rho }}_{th}^{E}\right)$$

by the additivity of von Neumann entropy for separable joint states of bipartite composite systems. For the final state of SE, we have

$$S\left({\hat{\rho }}_{i}^{{\mathrm{SE}}^{\prime }}\right)\leq S\left({\hat{\rho }}_{i}^{S^{\prime }}\right)+S\left({\hat{\rho }}_{i}^{E^{\prime }}\right)$$

by the subadditivity of von Neumann entropy for general joint states of such systems. The above three relations together yield

$$S\left({\hat{\rho }}_{i}^{S^{\prime }}\right)+S\left({\hat{\rho }}_{i}^{E^{\prime }}\right)\geq S\left({\hat{\rho }}_{i}^{S}\right)+S\left({\hat{\rho }}_{th}^{E}\right)$$

or

$$S\left({\hat{\rho }}_{i}^{E^{\prime }}\right)-S\left({\hat{\rho }}_{th}^{E}\right)\geq -\left[S\left({\hat{\rho }}_{i}^{S^{\prime }}\right)-S\left({\hat{\rho }}_{i}^{S}\right)\right].$$

Recognizing this final expression as the relationship $\Delta S_{i}^{E}\geq -\Delta S_{i}^{S}$ between the entropy changes of the system and environment, and substituting into (19), we have for the *i*-th process

(20) $$\Delta \langle E_{i}^{E}\rangle \geq -k_{B}T\ln\left(2\right)\Delta S_{i}^{S}.$$

With this, the average environmental energy change over the *M* processes is lower bounded as

$$\langle \Delta \langle E_{i}^{E}\rangle \rangle =\sum_{i}p_{i}\Delta \langle E_{i}^{E}\rangle \geq -k_{B}T\ln\left(2\right)\sum_{i}p_{i}\Delta S_{i}^{S}$$

which is the conditional energy bound (6).

### 3.2. Proof of the GLB (or Unconditional Energy Bound)

Now, to lower bound $\langle \Delta \langle E_{i}^{E}\rangle \rangle$ for unconditional operations, we first expand the average environment energy out as

(21) $$\begin{array}{c} \langle \Delta \langle E_{i}^{E}\rangle \rangle =\sum_{i}p_{i}\Delta \langle E_{i}^{E}\rangle =\left\{\sum_{i}p_{i}Tr_{E}\left[{\hat{\rho }}_{i}^{E^{\prime }}{\hat{H}}^{E}\right]\right\}-\langle E_{th}^{E}\rangle \\ =\left\{\sum_{i}p_{i}Tr_{E}\left[Tr_{S}\left[{\hat{\rho }}_{i}^{{\mathrm{SE}}^{\prime }}\right]{\hat{H}}^{E}\right]\right\}-\langle E_{th}^{E}\rangle \\ =\left\{\sum_{i}p_{i}Tr_{E}\left[Tr_{S}\left[{\hat{U}}_{i}{\hat{\rho }}_{i}^{\mathrm{SE}}{\hat{U}}_{i}^{†}\right]{\hat{H}}^{E}\right]\right\}-\langle E_{th}^{E}\rangle . \end{array}$$

Applying the linearity of the partial trace operation twice—first for the partial trace over E and then for the partial trace over S—Equation (21) becomes

$$\langle \Delta \langle E_{i}^{E}\rangle \rangle =Tr_{E}\left[\sum_{i}p_{i}Tr_{S}\left[{\hat{U}}_{i}{\hat{\rho }}_{i}^{\mathrm{SE}}{\hat{U}}_{i}^{†}\right]{\hat{H}}^{E}\right]-\langle E_{th}^{E}\rangle$$

and then

(22) $$\langle \Delta \langle E_{i}^{E}\rangle \rangle =Tr_{E}\left[Tr_{S}\left[\sum_{i}p_{i}\left({\hat{U}}_{i}{\hat{\rho }}_{i}^{\mathrm{SE}}{\hat{U}}_{i}^{†}\right){\hat{H}}^{E}\right]\right]-\langle E_{th}^{E}\rangle .$$

Now, to specialize this for unconditional operations, we require that all of the *M* state transformations are achieved via applicaiton of the same time-dependent process potential $V^{S}\left(t\right)$ to S and thus the same unitary operation $\hat{U}$ to SE:

(23) $${\hat{\rho }}_{i}^{{\mathrm{SE}}^{\prime }}=\hat{U}\left(\rho_{i}^{S}⊗{\hat{\rho }}_{th}^{E}\right){\hat{U}}^{†}.$$

Substituting ${\hat{U}}_{i}\to \hat{U}$ for all *i* in Equation (22) accordingly yields

(24) $$\langle \Delta \langle E_{i}^{E}\rangle \rangle =Tr_{E}\left[Tr_{S}\left[\sum_{i}p_{i}\left(\hat{U}{\hat{\rho }}_{i}^{\mathrm{SE}}{\hat{U}}^{†}\right)\right]{\hat{H}}^{E}\right]-\langle E_{th}^{E}\rangle .$$

Because the time-evolution operator is now independent of the pre-erasure state, the linearity of unitary similarity transformations can be applied to obtain

(25) $$\langle \Delta \langle E_{i}^{E}\rangle \rangle =Tr_{E}\left[Tr_{S}\left[\hat{U}\left(\sum_{i}p_{i}{\hat{\rho }}_{i}^{\mathrm{SE}}\right){\hat{U}}^{†}\right]{\hat{H}}^{E}\right]-\langle E_{th}^{E}\rangle .$$

The key observation at this point is that, for initial states of the form ${\hat{\rho }}_{i}^{\mathrm{SE}}={\hat{\rho }}_{i}^{S}⊗{\hat{\rho }}_{th}^{E}$, Equation (25) can be written as

(26) $$\langle \Delta \langle E_{i}^{E}\rangle \rangle =Tr_{E}\left[Tr_{S}\left[\hat{U}\left({\hat{\rho }}^{S}⊗{\hat{\rho }}_{th}^{E}\right){\hat{U}}^{†}\right]{\hat{H}}^{E}\right]-\langle E_{th}^{E}\rangle$$

where

(27) $${\hat{\rho }}^{S}=\sum_{i}p_{i}{\hat{\rho }}_{i}^{S}.$$

This is to say that, for unconditional operations, the environmental energy increase obtained via the straightforward average (5) over the *M* individual processes is equivalent to the energy increase of the environment for the single process

(28) $${\hat{\rho }}^{S}⊗{\hat{\rho }}_{th}^{E}\to {\hat{\rho }}^{{\mathrm{SE}}^{\prime }}$$

with the initial state of S described by the “surrogate” density operator ${\hat{\rho }}^{S}$ given by (27) (for which $Tr_{E}\left[{\hat{\rho }}^{{\mathrm{SE}}^{\prime }}\right]={\hat{\rho }}^{S^{\prime }}=\sum_{i}p_{i}{\hat{\rho }}_{i}^{S^{\prime }}$). Because of this mathematical equivalence, the desired unconditional erasure bound can be obtained by lower bounding the environment energy increase via Partovi’s inequality for the “surrogate” state transformation (28), which yields

(29) $$\langle \Delta \langle E^{E}\rangle \rangle \geq -k_{B}T\ln\left(2\right)\left[S\left({\hat{\rho }}^{S^{\prime }}\right)-S\left({\hat{\rho }}^{S}\right)\right].$$

It follows from the definition (10) of the Holevo information that

(30) $$S\left({\hat{\rho }}^{S}\right)=S\left(\sum_{i}p_{i}{\hat{\rho }}_{i}^{S}\right)=\chi^{S}+\sum_{i}p_{i}S\left({\hat{\rho }}_{i}^{S}\right)$$

(31) $$S\left({\hat{\rho }}^{S^{\prime }}\right)=S\left(\sum_{i}p_{i}{\hat{\rho }}_{i}^{S^{\prime }}\right)=\chi^{S^{\prime }}+\sum_{i}p_{i}S\left({\hat{\rho }}_{i}^{S^{\prime }}\right)$$

which finally yields

$$\langle \Delta \langle E_{i}^{E}\rangle \rangle \geq -k_{B}T\ln\left(2\right)\Delta \chi^{S}-k_{B}T\ln\left(2\right)\sum_{i}p_{i}\Delta S_{i}^{S}.$$

This is the generalized Landauer bound (8).

Several aspects of this proof warrant emphasis. First, nothing in the proof is tied to features of any particular model system belonging to the general class of systems considered here. The states used to encode logical inputs and outputs in any such system can be described by density operators and assigned a von Neumann entropy, whatever the structure and composition of the system and the specifics of the encoding scheme. Second, while the initial environment state is assumed to be in a thermal state with an assignable temperature *T*, no such “equilibrium” assumption is made for the environment state at any other time or for the system state at any time. Nor is there any kind of quasi-static assumption made about the processes that evolve the interacting system-environment composite. Third, nothing in the proof addresses or ensures the achievability of the GLB, so it is to be regarded as bound whose violation is precluded by physical law but whose achievability is not guaranteed or even addressed by the proof. 14

Fourth and finally, we emphasize that our proof of the GLB assumes that the state space of the system S remains unchanged throughout the duration of every one of the *M* processes. This is to say that all regions of the same system state space are accessible to the state of S at all times for all relevant processes, even when one or more of the process potentials $V_{i}^{S}\left(t\right)$ is such that occupation of some state subspaces is exceedingly unlikely at some times for some process potentials and initial states.

We mention this final point because a prominent objection to classical thermodynamic proofs of LP, due to Norton [6,15], stems from the construction and use of “illicit ensembles”—ensembles of states that are treated as canonical ensembles even though they are constructed from states defined on state spaces that are inaccessible to one another. This “mutual inaccessibility” is a consequence of the assumed impenetrability of partitions and pistons whose systematic insertion, displacement, and removal is stipulated in the idealized processes and classical systems used most widely in analyses of LP. The impenetrable partitions and leakproof pistons of these classical analyses are material analogs of infinitely high (and thus nonphysical) potential barriers. Because we consider only finite process potentials $V_{i}^{S}\left(t\right)$ in our proofs, and thus rule out the presence of any infinitely high potential barriers that could render state subspaces mutually inaccessible, concerns associated with the invocation of illicit ensembles do not apply to our proofs. Rather, our proofs uphold Landauer’s bound in very general quantum settings where the various physical states that encode information—even if arbitrarily well confined to state subspaces by arbitrarily high (but finite) potential barriers 15—retain access to the full system state space on which they are collectively defined. Thus, even if proofs of Landauer’s bound that invoke illicit ensembles are dubious, the absence of such nonphysical idealizations from our proofs rules out the possibility that Landauer’s bound is somehow an artifact of such idealizations. Rather, our proofs support the status of Landauer’s bound as a straightforward lower bound on the unavoidable dissipative cost of implementing logically irreversible operations via unconditional physical operations.

## 4. Summary

In this work, we have proved a generalized Landauer’s bound (GLB) using an approach that sidesteps many objections to existing proofs and demonstrations of Landauer’s Principle. In Section 1, we briefly discussed the ongoing controversy surrounding Landauer’s Principle as well as the sources and implications of this controversy. In Section 2, we described the very general physical setting to which the GLB applies and defined all relevant quantities, presented and discussed the GLB, and demonstrated specialization of the GLB to Landauer erasure of information encoded in distinguishable and indistinguishable states of a physical system and to ideal logically irreversible and reversible information processing operations. Along the way, we showed how the connection between logical and physical reversibility of erasure and logical operations is rooted in the conditioning of those operations—or lack thereof—on the states of the system that encode logical inputs and outputs. Then, in Section 3, we presented our proof of the GLB in detail and remarked on features of this proof that are germane to its interpretation and its immunity to some common objections.

The contributions of this work, beyond the GLB itself, are threefold:

The first contribution is our proof the GLB, which sidesteps most 16 of the prominent objections to standard proofs and demonstrations of Landauer’s bound while producing an even more general result. Because our bound applies to the “trial-averaged” energy change of the environment, it is immune to what is perhaps the most prominent objection to most proofs of LP: the controversial but nearly ubiquitous presumption that the entropies of ensembles of physical input states—ensembles that mix the input probabilities statistically characterizing the input source with the probabilities appearing in statistical descriptions of the physical input states themselves—can be regarded as ordinary physical entropies for the purposes of determining energy dissipation bounds. Our proof sidesteps this objection, upholding the GLB without making any such presumption. Significantly, bounds identical or equivalent to the GLB have been obtained using approaches that are based upon this presumption 17 or have otherwise appeared in the literature. 18 This vindicates the use of such approaches for obtaining energy dissipation bounds. Any related objections to proofs or demonstrations of LP that claim equivalence of thermodynamic entropy and information-theoretic entropy are similarly sidestepped in our proof: von Neumann entropies are used throughout the proof without presuming that they have any thermodynamic significance or using any relations that would require that they do have thermodynamic significance. Nor do we require that these entropies have any information-theoretic significance. The information measures that appear in our bounds—the Holevo information and the Shannon entropy—emerge naturally in the proof; they are not inserted “by hand” or presupposed to be in any way equivalent to this or that physical entropy.

The second contribution is clarification of connections between logical reversibility, physical reversibility, and conditioning. Recall that the conditional energy bound (6) includes only a single term: the (weighted) average of the system entropy changes for the *M* processes. Nothing ensures—but nothing precludes—the possibility that each of the *M* completed processes can be reversed by additional processes without irreversibly changing the environment energy. This is the case even for conditional erasure-by-reset and other logically irreversible information processing operations, since there is no information loss contribution in the conditional energy bound. Recall also that the unconditional energy bound (8)—the GLB—includes the conditional energy bound as one term and a term proportional to the information loss as well. Since the information-loss term, which is always nonnegative, is directly traceable to absence of conditioning, this identifies irreversible environmental heating with lack of conditioning of the processes that constitute an operation. However, since the information loss term vanishes for unconditional operations that do not lose information, our proofs of the conditional and unconditional energy bounds—taken together—point more specifically to the combination of information loss and lack of conditioning as the source of the irreversible system-to-environment energy transfer reflected in Landauer’s Principle. Indeed, while capturing the recognized connections between logical reversibility, physical reversibility, and conditioning of operations—i.e., the physical irreversibility of logically irreversible operations implemented unconditionally and the physical reversibility of logically reversible operations implemented conditionally—our results leave room for additional options. First, because they are not informationally lossy, the reversible physical implementation of *M*-to-*M* logically reversible operations via unconditional physical operations is not ruled out by our results. Second, even though they are informationally lossy, reversible physical implementation of *M*-to-*N* logically irreversible operations via conditional physical processes is not ruled out. Thus, our results leave room for physically reversible implementation of logically reversible and logically irreversible operations that are appropriately conditioned, since the dissipative information loss term is—for different reasons in the respective cases—absent from the applicable bounds.

The third contribution is the demonstration that variants of Landauer’s bound for erasure and logical operations—some obtained previously 19—all follow as specializations of the GLB and are thus unified here under a single framework. Specializing the GLB as required to treat erasure and logical operations—but no further—yields dissipation bounds for these operations that are considerably more general that the forms most familiar from the literature, allowing that the physical system states encoding different logical inputs may occur with different probabilities, may be imperfectly distinguishable from one another, and may have self-entropies that differ both from one another and from the self-entropies of the physical system states that encode logical outputs. Specializing these bounds further yields the more familiar forms, which typically apply for uniform input distributions, mutually distinguishable input states, and input and output or reset states with identical self-entropies. 

## Data Availability

Not applicable.

## Footnotes

1 The literature on Landauer’s Principle is vast, the original paper [1] having been cited well over six thousand times at this writing. For general background on Landauer’s Principle, including a sampling of historical and contemporary perspectives, see Refs. [2,3,4] and references therein.

2 Five examples of exchanges from the literature, spanning more than three decades, are collected in Ref. [5] (as Refs. [4]–[25] of that work).

3 There is an extensive literature on reversible computing, going back to the inaugural work of Bennett [7]. Contemporary understandings of the physical basis for reversible computing are discussed in detail in Ref. [8].

4 Such scenarios do not, however, accommodate systems like standard CMOS implementations of logic gates, in which physical encodings of the logical inputs persist in part of the system as the output states are generated and rendered in another part—i.e., in which the inputs are not physically overwritten by the outputs—and the outputs persist only as long as the inputs are retained. This does not suggest that there is no “Landauer cost” to information processing in standard CMOS; it is just to say that formal proof of Landauer’s bound in such a setting would require modification of the present formalism (not to mention inclusion of additional features such as external particle sources and sinks). Along with these additional formal complexities, any such proof would face the very same fundamental issues that arise—and that we seek to illuminate—within the simpler scenarios considered in this paper and in most studies of Landauer’s Principle.

5 The transformation of the local state of S resulting from this process, which generally is not unitary (as is evolution of the global state of SE), is described by a quantum operation or completely positive trace preserving map.

6 The adjective “conditional” recognizes that the process potential is conditioned upon the initial state, i.e., that if the initial system state is ${\hat{\rho }}_{i}^{S}$ then the process potential is $V_{i}^{S}\left(t\right)$.

7 The entropy differences $\Delta S_{i}^{S}$ that are averaged over in the bound (8) are changes in the self-entropies of the *M* individual encoding states—not any entropic measure associated with information encoded in the *M*-ary ensemble of states ${\hat{\rho }}_{i}^{S}$. The individual $\Delta S_{i}^{S}$ can be positive, negative, or zero—as can their ensemble average—whether information is gained, lost, or preserved in the process.

8 We note as an aside that, in conventional information processing devices, the primitive operations are performed unconditionally. In the reversible computing paradigm, however, elementary operations are performed conditionally.

9 One may naturally wonder if this external copy could be avoided by including, at an initial stage of the process, a measurement that would determine the inital state and then act accordingly. This does not solve the problem, however, as the outcome of the measurement must be rendered in a physical system outside of the device on which the measurement is performed, which constitutes creation of an external copy. (see exchange between Norton [6] and Ladyman and Robertson [12] on this issue, and [5] for related remarks.) This solution is problematic for other reasons as well, including possible lack of distinguishability of initial states by any measurement for some ensembles and, in some quantum settings, unavoidable modification of the initial states by the measurement process.

10 To be clear, *M* and *N* here are the respective numbers of distinct inputs and outputs to the information processing operation—not the numbers of digits used to represent the inputs and outputs in any particular number system. Consider, for example, the Boolean function implemented by a binary full adder, which has a 3-bit input and a 2-bit output. For this operation, the number of distinct inputs (or input vectors) is $M=2^{3}=8$ and the number of distinct outputs (or output vectors) is $N=2^{2}=4$. Note more generally that N≤4 for Boolean operations with 2-output bits, since some such operations—unlike the full adder—do not generate outputs corresponding to all $2^{2}=4$ possible combinations of two bit values.

11 Logically reversible operations include noiseless communication and memory.

12 The conditional entropy H(X|Y), which is the Shannon information in *X* that is not in *Y* or the input information that is lost in the input-output mapping from *X* to *Y*, generally is not equal to the difference H(X)−H(Y) between the Shannon entropies of correlated random variables *X* and *Y*. In general, they are related as H(X|Y)=H(X,Y)−H(Y), where H(X,Y) is the joint entropy of *X* and *Y*. However, for the mappings that describe the input-output relation for deterministic logical operations, H(X,Y)=H(X) so H(X|Y)=H(X)−H(Y).

13 Note that in Ref. [5] there are mathematical typesetting errors in Equations (9), (11) and (12), in the unnumbered equation before Equation (4) and the unnumbered equation between Equation (11) and Equation (12), and the inline equation appearing immediately after Equation (12). These typos, which did not effect the results of that work, have been corrected in all analogous expressions appearing in this work. The author thanks Sam Fletcher for bringing these errors to his attention.

14 Hence our terminological preference for “Landauer bound” over “Landauer limit” in this work. While they are used interchangeably, the latter seems more likely to carry a connotation of achievability.

15 Effective confinement of physical states to designated state subspaces is essential for reliable encoding of information in system states, but absolute confinement for all time is not required for reliable processing of encoded information. Rather, reliable information processing requires barriers high enough to confine system states to designated subspaces with exceedingly high probability over timescales that matter for the processing and intermediate retention of information. Indeed, the reliable encoding and retention of information in conventional computing devices is achieved via confinement of electrons to selected spatial regions (and thus state subspaces) by barriers that are penetrable—but that are “sufficiently impenetrable”.

16 Note that this paper does not address concerns related to the neglect of thermal fluctuations in proofs of Landauer’s Principle—another prominent point of contention—as the extent to which such concerns impact the present approach is unclear. A recent summary of such concerns, and a statistical mechanical treatment of Landauer’s Principle that aims to address them, can be found in [16].

17 These include the “referential approach”—see Equation (18) of [17] (with Equation (20) of that work) and Equation (18) of [18] (with Equations (1) and (6) of that work)—which is this author’s preferred approach to obtaining dissipation bounds in information processing scenarios for reasons described in Refs. [18,19].

18 For example, a bound identical in form to Equation (29) of this work—which is equivalent to the GLB—appears (and is tightened) in [20].

19 For example, the bound (12) for erasure with distinguishable encoding states appeared as Equation (34) in Ref. [17], and the bound (16) for ideal logical operations appeared as Equation (68) in Ref. [21] (appropriately specialized to ideal operations with efficacy measures $f_{L}$ and $F_{L}$ fixed at unity) and as Equation (11) in Ref. [5]. However, in these cases and all others of which we are aware—with the exception of [5] (the precursor to this work)—the now controversial use of surrogate density operators to represent ensembles of states in the determination of dissipation bounds was employed and presumed to be valid.
